# On the Proper Treatment of Consumption

**Published:** 1896-07

**Authors:** C. H. Wilkinson

**Affiliations:** Chief Medical Officer and Surgeon-in-Charge, St. Mary’s Hospital, Galveston, Texas


// TEXAS MEDICAL JOURNAL,

ESTABLISHED JULY, 1885.

PUBLISHED MONTHLY.—SUBSCRIPTION $2.00 A YEAR.

VOL. XII. AUSTIN, JULY, 1896. NO. 1.

### Original Contributions.

For the Texas Medical Journal.

### ON THE PROPER TREATMENT OF CONSUMPTION.

BY C. H. WILKINSON, M. D.,
Chief Medical Officer and Surgeon-in-Charge, St. Mary's Hospital,
Galveston, Texas.

PROBABLY, there is no disease flesh is heir to, that, when fully under headway, embodies so many pathological processes and other changes in the human system, as does consumption. Its ravages are by no means confined to deposits in the lungs, nor is it strictly speaking a local process, although in its insipiency it may appear so. There are carried in its train changes and conditions which would seem to entitle it to the distinctive appellation of a hydra-headed monster to the human family.

Starting in the lung, as a general rule, at least so in the southern portion of the United States, it speedily involves the blood; first, by septic, infection causing irritative fever, and scondly, by carbonization of that fluid (by excluding therefrom the proper amount of oxygen). Thus a double septicæmia is produced which soon debilitates the cardiac muscles to such an extent as to induce a state of tachycardia and general weakness of the body, that often render it difficult and extremely disagreeable for a patient to enjoy even moderate exercise.

Along with these disorders must be grouped one of the most discouraging conditions, both to the physician and the patient, namely anorexia, due undoubtedly to gastric catarrh, for; by destroying the functions of the stomach, the main avenue to treatment is cut off, and a slow but certain starvation awaits the victim, unless some substitute route to the sanguineous system can be reached by which the nourishment can be introduced, and waste of tissue averted. Add to these degenerations of the glands of the body, as of the liver, spleen, kidney, etc., and take into account the usually concommittant bronchitis, which so often makes the waning existence of the consumptive so extremely wretched; then add the sweats and other colliquative discharges due to the disease, and we will be able to appreciate what we have to deal with in an ordinary case of phthisis.

I make no mention of hemorrhage from and condensation (pneumonic processes) of the lungs, with collapse of the air cells, as these are so closely connected, geographically, with the initial process as in reality to be a part and parcel of its nature. But it is very necessary to understand that no treatment can be considered successful which leaves behind any probability of the one, or remnant of the other; for so sure as a consumptive is dismissed from treatment with the slightest vestige of tuberculosis left behind, just so certain will the disease break out with renewed activity in a very short time thereafter.

Briefly scanning, then, by way of premise, the many destructive processes at work in cases of consumption when fairly started, does it not appear presumptuous and even unwise to proclaim "Eureka" to any single remedy for the treatment of such a disease? It certainly appears so to me; and this fact of the utter unreliability of so-called specifics in consumption has been fully substantiated by my observation for over twenty years, in one of the largest hospitals in the southwest. For twenty-five years I have been a close observer of all the forms of this disease, and scarcely anything of promised merit, as a means of relief, has escaped my employment. What I have not personally tested, I have watched its experimentation in the hands of others. As a result of such employment and observation, I have come to the conclusion that, at present, there is no specific known to our profession for the cure of consumption. This, however, might reasonably have been surmised from a careful consideration of the nature and usual complications of phthisis. Being, as it were, a compound disease, it stands to reason that the remedies for its relief must also be of a compound nature.

For its relief, I repeat, for phthisis, in my judgment, can be relieved in its early stages just as readily as incipient and reachable carcinoma can, while on the other hand, each is about as deadly as the other when it has reached the latter stage.

Any one who has watched the destruction of vegetable life by micro-organisms, as for instance, upon trees, shrubs and other plants, and witnessed the counter-operation of germicides, must have learned an object lesson which might easily be applied to human life.

The horticulturist has solved the problem of his success by using many of our most common remedies, while only a few years since the application of such remedies was unknown, and thousands upon thousands of valuable vegetable lives perished through such ignorance. In phthisis we have the micro-organisms at their deadly work, and our most natural remedy against such organisms must be a germicide applied, if possible, *directly* to them. Which of them is the most applicable is now the practical question. Could we but spray our patients' lungs with Paris Green or London Purple (in the incipient stage) without killing them, the question of curing consumption would be solved with us. We must seek, however, some other agent, less deadly to the human race, but equally destructive to the bacillus of this disease. Such an agent I believe is found in creasote.

There is no doubt as to the destructiveness of this drug upon the germs of phthisis. You have only to use it appropriately for a few days in order to demonstrate this fact. Under the creasote treatment bronchial irritation is almost immediately relieved, and the cough subsides as though by magic. If you examine the sputum of your phthisis patient at the beginning of your treatment (as you always should), and then again a fortnight later, it will be observed that the germs have markedly diminished in number. In the same time the appetite will have improved, and the body weight increased.

Creasote is a drug whose virtues I can not praise too highly in consumption. Under its influence I have seen patients raised up from what was honestly believed to be their death beds, and resume a life of useful occupation. Surely, any remedy which can do so much in the latter stages of consumption, must be a valuable adjunct to other remedies in the beginning of that disease. In order to obtain its full effects however, creasote should be given both internally and by inhalation. There is a certain amount of technique to be observed in its employment which, if neglected, will only lead to disappointment in treating our cases.

Given by inhalation, we should endeavor to maintain as steady and constant a supply of it as possible. Its use by day and night would be the preferable manner, were it not for interference with other remedies to be referred to presently. One daily seance in the "smoke room" is not sufficient, but three or four should be submitted to, each lasting thirty minutes. Nor should the vapor thereof be used too strong. If irritating or offensive to the patient its strength should be diluted, or its employment discontinued for a few days. I have a room arranged and properly located where I have my patients inhale the fumes of creasote from a Coulter's vaporizer for the space of forty minutes three times a day, and longer when necesssary. During such inhalation the patient should be made to take full and deep respiration, otherwise the vapor would not find access to all the air cells. An attendant, therefore, should always be on hand to see that every minutia of the inhalation process was being carried out. I have on many occasions had vaporizers placed in the sick rooms of my cases, and the odor of creasote was alwas present day and night. This is an excellent method of employing it, as the influence of the remedy upon the disease is practically continuous. But it must not be expected to cure such a disease as consumption by inhalation alone; especially in a case where the advanced stages have been reached. This is obvious from the fact that creasote can only interrupt the existence of bacilli which come in personal contact with its fumes. When the pathological factor is imbeded in the pulmonary exudates, encysted as it were by plastic material, and away from the open air cells, no vapor of any kind can reach them.

For this contingency which exists in the majority of cases which come under the observation of the physicians, I give creasote *internally*. Unfortunately the acrid nature of this drug precludes its administration in other than the smallest doses. There is, however, an excellent preparation of creasote and carbonic acid in combination, upon the market, which is acceptable to patients in teaspoonful doses, but this preparation is costly, and is covered by patent, rendering its use impracticable for the majority of cases. My method of using the drug internally is in the form of an emulsion, combining it with whisky and petroleum as follows:

℞ Mass. petrolei ........................... 4 ounces

Creasoti opt ................................. 2½ drams

Ovi vitellus ................................. 2 —

Syrup simp ................................... 1½ ounces

Spt. frumenti, to make .................. ½ pint

Mix. S. Dose, a dessertspoonful in a gill of sweet milk at 8, 12, 4 and 8 o'clock daily. By nurse.

This combination can be administered continuously for months without detriment to the patient, and can be taken alone or in sherry, as desired.

The petroleum used in this emulsion is regarded as a most valuable adjunct. I have employed it in other formulas for over twenty-five years, and have so far failed to find a single case of phithisis that was not benefited by its use. It certainly stimulates digestion, aids nutrition, is undoubtedly a germicide of high power, and exercises a marked influence over respiratory diseases. Its combination with the creasote and other ingredients just cited, renders it an ideal "Lung Food," and in reality I have found it such in every case so far using it. But a disease like the one under consideration can not be successfully treated by drugs alone. As stated before, consumption is a compound disorder, and the victims of which almost invariably perish by starvation. The proper treatment, therefore, of such must be compound in its nature. Much stress of late has been placed upon the "climatic treatment" of this disease, and, indeed, pure air in a bracing temperature has often staid the ravages of phthisis for a considerable time. But you can not cure your consumptives upon air alone. It is an indispensable auxiliary, I admit, and must be obtained in its purest and freshest stote in treating these cases. So important, indeed, has this atmospheric element become of late, that many of the old time traditions concerning the climatic treatment of phthisis have been brushed away, and now the desideratum is that climate which admits of the greatest amount of outdoor exercise for patients with consumption, whether it be mountainous or seashore, dry or moist.

Besides the administration of drugs, as before mentioned, fresh air and moderate exercise must be enjoined upon our patients. They must be made to walk, ride, run, anything to keep them going in the open air at all times, when not interfering with their natural rest and the balance of their regular treatment.

Moping is sure death to consumptives, as is self-centered reflection. They must be made to get out and stir in some systematic manner, and the three methods of exercise just mentioned are the most suitable for such cases.

Pulmonary Gymnastics.—By this I mean such exercise as will develop the pectoral muscles, expand the thorax, and inflate the air cells. Denison's manometer is perhaps the most suitable instrument for this purpose, on account of its cheapness and simplicity; and also as it affords a faithful index of a patient's improvement. An excellent method of chest exercise is obtained by the use of Hulbert Bros. & Co.'s "Home Exercising Machine," which, with little effort on the part of the patient, brings into play all the muscles of the upper extremity. The use of these two will suffice for any ordinary case. They should be used morning and evening along with the inhalations and medication prescribed, and a faithful nurse or attendant should be charged with the duty of seeing that the entire system of treatment is being punctually carried out.

Feeding in Consumption.—I now arrive at one of the most important divisions of my subject. For after all, no treatment of any kind can succeed in this disease if the pabulum, the nourishment of our patients, is allowed to become deficient. The waste of tissue being rapid and enormous, the supply of food must likewise be maintained even to the limits of toleration. Food, but suitable food, must be given, oftener than usual. I have my patients fed every four hours in the day, preferably at 8, 12, 4 and 8 o'clock. The general make up of such sustenance should be nutritious and digestible, no matter what the special article should be. In this connection, I will state that mistakes have often been made by us in our early practice, by sending cases off to climatic resorts, where air was plentiful, but beef, the king of nutrients in consumption, was rarely ever seen. The consequence has been that, in the majority of such instances, our patients have been starved to death away from home. The gastric catarrh hitherto alluded to, is the greatest enemy against the patient, of all the forces arrayed against him, and requires a constant warfare on our part to baffle his designs. The manner of feeding often becomes repulsive when pushed too far, therefore change and delicacy are always to be observed, bearing in mind that an important article of food should not be offered too often, lest it disgust the patient. Beef, in some shape or other, should be given daily; if not in the usual dietary form, then in the way of extracts, etc. I have often administered defibronated beef blood by enema. From four to six ounces can be given this way every night and morning, and given in this manner proves an excellent remedy. The blood should be first warmed to about 140° F. and allowed to cool to 98°, or thereabouts, and should only be taken from healthy animals. I have often seen splendid results from the use of such enemas, the pulse falling and the appetite and strength increasing under its use day by day, until the patients were enabled to retain and enjoy their usual food.

This, then, briefly covers my treatment of consumption as ordinarily met with here in Galveston, and it will be seen that it partakes of the *medicinal*, the *mechanical*, and the *dietetic*. My results have been so satisfactory under it, even in the latter stages of the disease, that I do not hesitate to assert that, where the plan is adopted in the first stage of phthisis, and persevered in, the majority of such cases can be cured.

Unfortunately, however, to carry out so methodical a line of treatment is practically impossible to many of our cases. As a rule, patients can not be cured at home, for the simple reason that at home the discipline so necessary to be practiced in their cases is rarely ever practicable. They require system, vigilance, intelligence and effort in their treatment, and this acknowledgment brings me to the consideration of the sanitarium.

If you have carefully followed me through the various stages of my plan of treatment in consumption, you will readily admit that, in order to carry out its manifold provisions, each department of which is vitally important in order to obtain its benefits, some suitable establishment should be provided for the successful prosecution of the plan. Were such cases assigned to properly constructed and managed homes, where an intelligent and painstaking discipline could be maintained over them at all times, and where the treatment outlined above could be carried out in its entirety, the mortality of phthisis, which now excells that of any other ailment on the face of the earth, would be cut down, and thousands of our suffering human beings would be rescued from untimely graves. When we behold temples erected for the blind, the deaf, the insane, and the crippled, does it not produce a natural wonder that consumptives, the most unfortunate, or rather, the most universal of any other class of suffering humanity, should be neglected as they are? The sanitarium, then, is the proper place to treat consumptives, where all the details of a special treatment can be carried out, and where the most approved methods of dealing with their cases can be regularly and patiently indulged in. These sanitariums should be constructed all over the country, keeping in mind the cardinal principle laid down in the body of this article, that the place to have our patients treated is where they can enjoy the greatest number of hours in the open air, all the year round. Right here in Texas we have the finest climate in the world for consumptives, and Galveston, on account of her salubrity, her ephemeral winters, and breeze-tempered summers, her singular freedom from malaria, and her capacity for furnishing diversion for health seekers, is peculiarly fitted for the location of such a place for tubercular patients. In fact any locality will suffice for such purpose, whether it be by sea shore or on mountain top, provided the regime directed before shall be fully observed. Conducted upon proper principles, they would be successful in their mission, and consequently well patronised by seekers after health; for men will go anywhere, and undergo any sacrifice, in order to stay the remorseless current of consumption. I know of no better or wiser use for money to-day, than its investment in a sanitarium, where so much benefit can be safely guaranteed for phthisis as can be done under the plan I have just briefly outlined. Who, then, will take the initiative in such an enterprise? Who will lead in this noble work of charity and humanity?

